# Integration of ANN-GA optimization and multi-scale mechanistic analysis for ultrasound-assisted enzymatic extraction of flavonoids from *Cortex Mori*^☆^

**DOI:** 10.1016/j.ultsonch.2026.107906

**Published:** 2026-05-24

**Authors:** Haixia Che, Jiawen Li, Huiru Fu, Yue Hu, Di Gao, Zejun Li, Qiao Li, Yu Xiao, Yue Zhou, Mingming Lian, Qian Li

**Affiliations:** a Department of Pharmaceutical Analysis and Analytical Chemistry, College of Pharmacy, Harbin Medical University, No. 157 Baojian Road, Nangang District, Harbin 150081, Heilongjiang, PR China; b Harbin Med Univ, Coll Pharm, Key Lab Gut Microbiota & Pharmacogen Heilongjiang, Harbin 150081, Heilongjiang, PR China; c Department of Pharmaceutical Chemistry, College of Pharmacy, Harbin Medical University (Daqing), Heilongjiang 163711, PR China; d Department of Inorganic Chemistry and Physical Chemistry, College of Pharmacy, Harbin Medical University, Heilongjiang 150081, PR China; e Department of Pharmaceutical Analysis, College of Pharmacy, Harbin Medical University (Daqing), Heilongjiang 163711, PR China

**Keywords:** *Cortex Mori*, Response surface methodology, Artificial neural network-genetic algorithm, Molecular dynamics simulations, UHPLC-HRMS, Bioactivities

## Abstract

*Cortex Mori* (CM), a valuable medicinal plant with abundant flavonoids, exhibits diverse biological and pharmacological activities. This work aims to develop an environmentally friendly and efficient ultrasound-assisted enzymatic extraction (UAEE) strategy for extracting flavonoids from CM, while also elucidating the mechanism underlying this process, characterizing major flavonoids and evaluating their bioactivities. First, UAEE was utilized for flavonoid extraction, and the critical parameters were determined using Plackett-Burman design (PBD), followed by optimization by integrating response surface methodology (RSM) with the Artificial Neural Network (ANN)-genetic algorithm (GA) approach. In addition, FT-IR, SEM and molecular dynamics simulations (MDS) were employed to elucidate the extraction mechanisms. UHPLC-HRMS was utilized to identify flavonoids in CM extracts, and the antioxidant and anti-proliferation activities were assessed. Results demonstrated that the ANN-GA model optimization outperformed RSM, yielding a maximum total flavonoid content (TFC) of 21.18 ± 0.94 mg/g under optimized conditions using an ultrasonic bath system, with ultrasonic parameters of 40 kHz and 200 W, combined with a solid-to-liquid ratio of 1: 24 g/mL, ultrasonic temperature of 55 ℃, ethanol volume fraction of 67 % and ultrasonic time of 60 min. Mechanistic analysis revealed that ultrasound promoted structural disruption of cell walls, altered intermolecular interactions, and enhanced solvent accessibility, thereby facilitating enzyme-assisted flavonoid release. A total of 41 flavonoids were tentatively characterized in CM extracts. CM extracts exhibited antioxidant capacity, including ABTS^+^ scavenging activity (0.8940 ± 0.0010 mmol/L), DPPH scavenging activity (96.78 ± 4.28 %) and ferric reducing antioxidant power (FRAP, 1.3074 ± 0.1234 mmol/L). CM extracts also showed antiproliferative activity against SW620, 4 T1, A2780, LOVO, and MCF-7 cell lines. Overall, this research provides multi-scale optimization strategies and mechanistic insights into the UAEE process, and offers theoretical guidance for the efficient utilization of CM and other botanical resources.

## Graphical abstract

**Figure:**
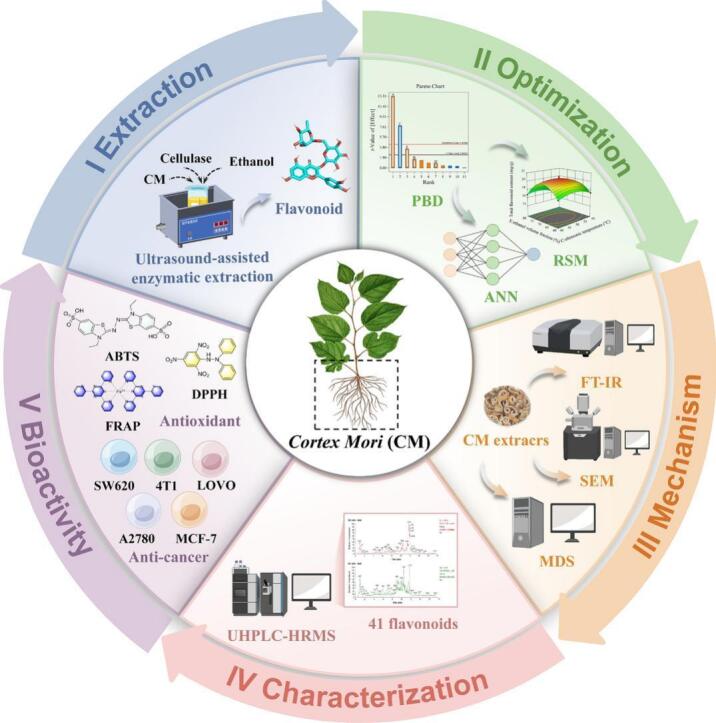

## 1 Introduction

*Cortex Mori* (CM) is derived from the dried root bark of *Morus alba L* and originates from China, with a long history of clinical application due to its diverse pharmacological activities [1], [2], [3]. It is reported that the clinical application value of CM is largely attributed to its abundant flavonoid compounds, such as sanggenon C, morusinol, and morusin, which are recognized as the principal bioactive constituents [4], [5], [6]. Nevertheless, conventional flavonoid extraction techniques, such as cold soaking and thermal reflux, typically require prolonged processing times and substantial consumption of organic solvents, leading to heat-related deterioration of sensitive compounds and excessive resource consumption [7]. Thus, it is of great importance to establish more effective extraction strategies for maximizing the yield, purity, and biological activity of flavonoid compounds from CM.

Ultrasound-assisted extraction (UAE), an environmentally favorable extraction technology, has attracted widespread attention due to its cost-effectiveness, time efficiency, high yield, and compatibility with thermolabile components [8]. The “cavitation effect” generated by ultrasound can disrupt the integrity of plant cell structures, thereby improving solvent accessibility and accelerating the diffusion of target compounds into the extraction medium [9]. More importantly, the UAE provides a more stable and suitable temperature for compound stability and enzyme activity, which could minimize the formation of undesirable by-products and ultimately elevate product quality [10]. Given these advantages, researchers commonly utilize sonication approaches to obtain flavonoids from diverse botanical materials [11]. Therefore, to obtain a higher yield and maintain good biological activity, this study intends to employ UAE to extract flavonoids from CM.

Recently, the application of mathematical and statistical tools has greatly advanced the development of extraction process optimization, enabling data-driven parameter selection, increasing yield and efficiency while reducing experimental workload, time, and material consumption [12]. Appropriate extraction parameters can considerably affect the content and composition of active compounds. Hence, a comprehensive optimization of the extraction procedure is indispensable. RSM and ANN are two common methodologies for the prediction and optimization of extraction parameters, which have attracted growing interest in extracting active ingredients from natural plants [13]. As a robust statistical tool, RSM enables the determination of optimal operational conditions and the evaluation of factor interactions with a limited number of experiments, providing reliable and precise results to guide subsequent optimization [14]. ANN is a non-linear computational modeling technique inspired by biological neural systems [15]. In comparison to RSM, ANN is advantageous in handling highly interactive or complex relationships, benefiting from its strong nonlinear fitting capability [16]. Genetic algorithm (GA) is a population-based optimization model that emulates natural evolution to find optimal solutions [17]. Previous studies have demonstrated that the ANN-GA model outperforms traditional regression-based techniques in various areas [18], [19]. However, to date, no study has optimized the extraction process parameters of flavonoids from CM using ANN-GA and RSM models.

This study employed a novel strategy of extracting flavonoids from CM using the UAE method. After screening critical factors that significantly influence the extraction process through PBD, a combination of RSM and ANN-GA was utilized to find optimal extraction parameters. Additionally, a multi-faceted extraction mechanism analysis was conducted by integrating FT-IR, SEM and MDS. UHPLC-HRMS was applied to characterize flavonoid compounds in CM extracts. Finally, the antioxidant and anti-cancer activities of CM extracts were evaluated in vitro. In summary, this work provides valuable insights for establishing an efficient approach for extracting flavonoids from CM, offering theoretical support for the utilization of CM within the medical and healthcare fields.

## 2 Materials and methods

### 2.1 Materials and chemicals

CM was purchased from Heilongjiang Hongci Pharmaceutical Co., Ltd (Harbin, China). Ethanol was from Fuyu Fine Chemical Industry Co., Ltd (Tianjin, China). Cellulase was obtained from MCE (Shanghai, China). The detailed information of materials and reagents is listed in Table S1.

### 2.2 Extraction of CM

#### 2.2.1 Cold soaking-assisted extraction (CAE)

CM powder (1.0 g) was added to 20 mL of 60 % ethanol, thoroughly mixed, weighed, and sealed. After a 60 min soaking period, any solvent evaporation was compensated by adding 60 % ethanol to restore initial weight. To isolate the liquid fraction, the extracts underwent a 10 min centrifugal separation (8000 rpm).

#### 2.2.2 Heating reflux extraction (HRE)

1.0 g of the sample and 20 mL of 60 % ethanol were added to the reflux apparatus. The mixture was heated to reflux for 60 min. The evaporated solvent was replenished by adding extraction solvent until the initial weight was reached. The liquid phase was isolated by centrifugation (8000 rpm, 10 min)

#### 2.2.3 Ultrasound-assisted extraction (UAE)

The UAE of TFC in CM was carried out utilizing a laboratory-scale ultrasonic cleaner (KS-250DE, Ultrasonic frequency: 40.0 kHz, Kunshan Jielimei Instruments Co., Ltd, Suzhou, China). The bath working volume was kept constant (2.5 L), and the applied power was used as a relative indicator of ultrasonic intensity under these conditions. 1.0 g of CM powder was combined with 20 mL 60 % ethanol in a beaker. After treatment in an ultrasonic cleaner (40 kHz, 200 W, 50 ℃) for 60 min, the samples were cooled, weighed, and any weight loss was compensated. The mixture was centrifuged at 8000 rpm for 10 min. The supernatant was collected for subsequent analysis.

#### 2.2.4 Enzyme-assisted extraction (EAE)

CM powder (1.0 g) and cellulase (20 mg) were added to 20 mL of 60 % ethanol (pH = 4.6). The mixture was maintained in a thermostatic bath at 50 ℃ for 60 min, and then heated in boiling water (100 °C) to inactivate the enzyme. After compensating for any weight loss, the extracts were centrifuged (8000 rpm, 10 min), and the supernatant was collected.

#### 2.2.5 Ultrasound-assisted enzymatic extraction (UAEE)

1.0 g of the CM powder, 20 mg cellulase, and 20 mL of 60 % ethanol (pH = 4.6) were mixed and treated according to the same parameters as UAE. The extracts were centrifuged (8000 rpm, 10 min) to obtain supernatant.

### 2.3 Determination of TFC

The TFC in CM extracts was determined as previously described with modifications^20^. Briefly, the extracts and 5 % sodium nitrite were thoroughly mixed, then stood for 6 min. Subsequently, 10 % aluminum nitrate was added, mixed, and stood for another 6 min. After adding 4 % sodium hydroxide solution, the mixture was diluted to 10 mL with blank solvent, and then reacted for 15 min. At a wavelength of 510 nm, the absorbance of the resulting mixture was measured. The measurement of TFC was calculated based on a standard curve [*C* = 5.8833*A* + 0.0031, *R^2^* = 0.9999] and *Eq.* (1).

(1) $$\mathrm{Yield}=\left(C\times V\times N\right)/M$$

where C is sample concentration (mg/mL), V represents the volume of CM extracts (mL), N represents the dilution ratio, and M represents CM powder weight (g).

### 2.4 Single-factor experiment

To optimize extraction process for obtaining the highest TFC, single-factor experiments were conducted as follows: solid-to-liquid ratio (1: 10–1: 35 g/mL), ultrasonic time (15–90 min), ultrasonic temperature (30–70 ℃), ultrasonic power (125–250 W), ethanol volume fraction (30–80 %).

### 2.5 Plakett-Burman design

The pivotal factors influencing the TFC from CM were screened using PBD among five factors, including solid-to-liquid ratio (A), ultrasonic time (B), ultrasonic temperature (C), ultrasonic power (D), and ethanol volume fraction (E). The optimization factors and related levels are shown in Table S2. The first-order polynomial model is described in *Eq.* (2)

(2) $$Y=\beta_{0}+\sum_{i=1}^{k}\beta_{i}X_{i}$$

Where *Y* is the response value, *β_0_* is the intercept, *β_i_* is the linear regression coefficient, *X_i_* represents the coded independent variable, and *k* represents independent variables’ number.

### 2.6 Response surface design

Based on the PBD experiment, three significant factors were further optimized to explore the interplay of these factors on TFC. The BBD (Box-Behnken Design) test was performed at three-factor and three-level using Design-Expert 13.0 software (Table S3). Multiple regression analysis was carried out to evaluate the effects of three independent factors on TFC. The regression equation was *Eq.* (3)

(3) $$Y=\beta_{0}+\sum_{i=1}^{k}\beta_{i}X_{i}+\sum_{i=1}^{k}\beta_{\mathrm{ii}}X_{i}^{2}+\sum_{i=1}^{k}\times \sum_{j=i+1}^{k}\beta_{\mathrm{ii}}X_{i}X_{j}$$

Where *Y* represents the response value, *β_0_* is the constant coefficient, *k* is the number of independent variables, *X_i_* and *X_j_* are the coded independent variables, and *β_i_*, *β_ii_*, and *β_ij_* are the coefficients of the linear, quadratic, and interaction terms, respectively.

### 2.7 BP-GA neural network analysis

This study employed an artificial neural network (ANN) to optimize the extraction process of flavonoid compounds based on 51 samples obtained from Latin hypercube sampling. A three-layer backpropagation (BP) neural network architecture was constructed using MATLAB R2022a software. The input layer contained three factors, including solid-to-liquid ratio, ultrasonic temperature, and ethanol volume fraction, while the output layer was TFC. The number of neurons in the hidden layer was determined after repeated attempts until the maximum regression (R) and the minimum mean squared error (MSE) were achieved. The data were randomly assigned into three subsets, including training (70 %), validation (15 %) and testing (15 %) and set, and normalized for training. The training parameter settings were as follows: maximum training iterations were set to 1000, learning rate was 0.01, and error threshold was 10^-6^. The ANN model was optimized using a genetic algorithm (GA) with a maximum iteration of 50, a population size of 30, a crossover probability of 0.7, and a mutation probability of 0.15. This optimization process continued until the optimal conditions were identified.

### 2.8 Mechanism analysis of UAEE

#### 2.8.1 FT-IR spectroscopy

Dried samples before and after UAEE process were mixed with KBr (1: 100) and finely ground. Spectra were acquired using a spectrometer (Shimadzu, Japan) at a wavenumber range of 400–––4000 cm^−1^.

#### 2.8.2 SEM analysis

Dried samples treated at different times were mounted onto conductive adhesive and coated with a thin gold layer prior to observation. Morphological features were then examined using SEM (HITACHI, Japan) at an accelerating voltage of 10 kV. Micrograph records were acquired at magnifications of 500 × and 2500 × .

#### 2.8.3 MDS analysis

MDS were conducted in Materials Studio 2020 to clarify the mechanism for extracting TFC from CM. Models of ethanol, water, and sanggenon C were constructed and optimized using the Forcite module with the COMPASS III force field, with atomic charges assigned via charge equilibration. An amorphous cell system (67 % ethanol–water-sanggenon C) was generated, and energy minimization was performed before simulation. All simulations were carried out under fixed conditions (328 K, 1 atm) using periodic boundary conditions, with trajectory snapshots saved every 1 ps for subsequent analysis.

### 2.9 UHPLC-HRMS analysis

The chromatographic separation was carried out utilizing a UHPLC system (Vanquish, Thermo Fisher Scientific, USA). Thermo Orbitrap Exploris120 Mass Spectrometer (Thermo Fisher Scientific, USA) was used for MS analysis. Data were collected using the data-dependent acquisition (DDA) mode (Full Scan-ddMS2). The detailed UHPLC-HRMS parameters and gradient elution program were listed in Table S4 and S5.

### 2.10 Assays of antioxidant activity

DPPH radical scavenging assay was carried out as previously reported [21]. CM extracts and DPPH solution were mixed (1: 1, v/v). After 30 min incubation under light-protected conditions, the absorbance was determined at a wavelength of 517 nm. The DPPH scavenging rate was calculated according to *Eq.* (4).

(4) $$\text{DPPH scavenging ability}\;\left(\%\right)=\left[1-\left({\text{A}}_{1}-{\;\text{A}}_{2}\right)/{\text{A}}_{0}\right]\times 100\%$$

where A_0_, A_1,_ and A_2_ represent the absorbance of DPPH solution, mixture solution, and sample, respectively.

ABTS^+^ radical scavenging assay was performed with an antioxidant capacity assay kit. According to instructions, the mixture of samples, ABTS working reagent, and reaction solution was incubated for 6 min while maintaining the system at room temperature and detected at 405 nm.

The ferric reducing antioxidant power (FRAP) of CM extracts was assessed in accordance with the instructions of the assay kit. 5 μL of samples and 180 μL of FRAP working solutions were combined. After incubation at 37 °C for 5 min. Absorbance was recorded at 595 nm.

### 2.11 Antitumor activity

Cells were cultured with Dulbecco’s modified eagle medium (DMEM) containing 10 % Fetal Bovine Serum (FBS). Following the preparation of a cell suspension at an appropriate concentration, the cells were transferred into 96-well microplates and incubated overnight (37 ℃, 5 % CO_2_). The cells were exposed to graded concentrations of CM extracts and maintained under these conditions for 24 h. For MTT assay, MTT solution (5 mg/mL) was incubated with cells for 4 h. After removing the supernatant, the precipitate was dissolved using DMSO, thoroughly mixed and absorbance was measured at 490 nm.

### 2.12 Statistical analysis

All data were expressed as mean value ± SD. Statistical comparisons were conducted through one-way analysis of variance (ANOVA) and differences were regarded as significant when *P* value was below 0.05. The results were visualized using GraphPad Prism 8 and Origin 2024.

## 3 Results and discussion

### 3.1 TFC in CM of various extraction methods

**Fig. 1:**
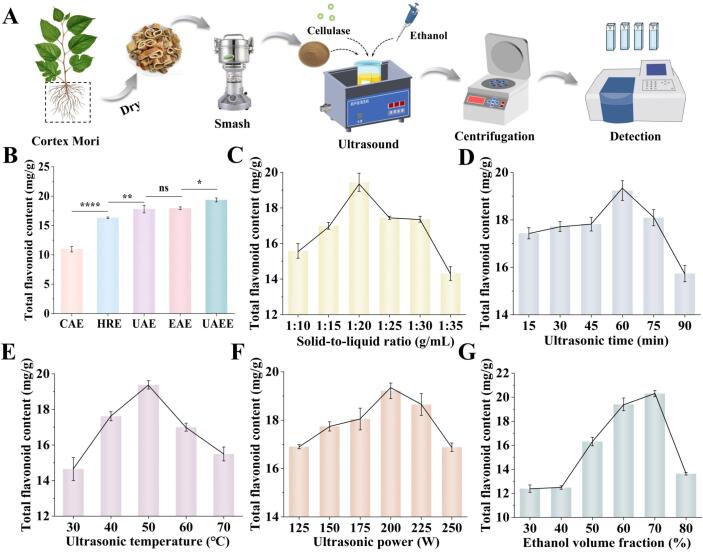
Influence of various extraction approaches and single factor on TFC (A) The flowchart of ultrasound-assisted enzymatic extraction. (B-G)The effect of different extraction parameters on total flavonoid content. n = 3, data represent mean ± SD, *P < 0.05, **P < 0.01, ****P < 0.0001, ns, not significant.

As presented in Fig. 1B, the TFC of CM was determined using five extraction methods, including CAE, HRE, UAE, EAE, and UAEE. It was clearly demonstrated that the TFC obtained by EAE, UAE, and UAEE was significantly higher than that obtained by the conventional CAE and HAE methods. Among these, UAEE exhibited the highest extraction efficiency, yielding 19.35 ± 0.33 mg/g of TFC (Fig. 1A and 1B), which was approximately twice that of CAE. This enhancement could be attributed to ultrasonic cavitation, which disrupted the integrity of plant cell structures, improved solvent and enzyme penetration and accelerated the release of intracellular flavonoids [22]. Considering economic feasibility, environmental sustainability, and extraction efficiency, UAEE was identified as the optimal extraction method.

### 3.2 Analysis of single-factor experiment

#### 3.2.1 Solid-to-liquid ratio

The effect of the solid-to-liquid ratio on the TFC in CM extracts was investigated from 1: 10 g/mL to 1: 35 g/mL. As displayed in Fig. 1C, following the rising solid-to-liquid ratio, the content of CM flavonoid exhibited an increasing trend, ultimately reaching a peak value at 1: 20 g/mL, corresponding to TFC of 19.44 ± 0.51 mg/g. However, the TFC in CM extracts displayed a decreased trend beyond this ratio. In general, a lower solid-to-liquid ratio led to insufficient contact between the solvent and solid. Moreover, a lower concentration gradient was unfavorable for the extraction of TFC [23], while increasing the solid-to-liquid ratio could effectively improve this situation. However, an excessively high solid-to-liquid ratio reduced the ultrasonic energy density, thereby reducing the penetration ability of the ultrasonic system [24]. Moreover, a higher solid-to-liquid ratio may cause unnecessary solvent consumption. Therefore, 1: 20 g/mL was considered the optimal solid-to-liquid ratio.

#### 3.2.2 Ultrasonic time

The effect of ultrasonic treatment time on the extraction of TFC from CM was evaluated over a range of 15 to 90 min. Fig. 1D illustrated that the TFC increased progressively as the ultrasonic treatment time was extended from 15 to 60 min, and reached the highest value (19.24 ± 0.42 mg/g) at 60 min. While further increase in ultrasound time led to a decline in TFC of CM. This phenomenon could be attributed to the cumulative ultrasonic and thermal effects generated over time, which promoted the release, diffusion, and dissolution of flavonoid compounds [25]. Nevertheless, prolonged ultrasonic exposure may cause hydroxyl radicals’ generation and heat accumulation, leading to chemical and thermal degradation of flavonoids [26], [27]. Accordingly, an ultrasonication duration of 60 min was determined to be the ideal condition for extracting TFC from CM.

#### 3.2.3 Ultrasonic temperature

Optimization of ultrasonic temperature is crucial for maximizing flavonoid yield, particularly in an enzyme-assisted extraction system. As the ultrasound temperature increased from 30 to 50 ℃, the TFC of CM rose from 14.64 ± 0.65 mg/g to 19.38 ± 0.24 mg/g. Beyond this threshold, a continued increase in temperature resulted in a gradual reduction in TFC (Fig. 1E). Lv et al. also observed a similar phenomenon in extracting polyphenols from hawk tea [28]. The possible reason may be that elevated temperatures reduced viscosity and surface tension while increasing vapor pressure, thereby promoting the formation of cavitation bubbles, which facilitated cell wall disruption and enhanced the diffusion of flavonoids into the solvent. However, excessively high temperature was not conducive to the propagation of ultrasound due to the continuous increase in vapor pressure within the microbubbles [29]. Moreover, 50 ℃ was within the optimal temperature range for cellulase activity, whereas excessively high or low temperatures impaired enzymatic hydrolysis and thereby reduced flavonoid yield [30]. Therefore, an extraction temperature of 50 ℃ was selected to reduce energy consumption while ensuring excellent extraction efficiency.

#### 3.2.4 Ultrasonic power

The influence of ultrasound power on the extraction of flavonoids from CM was also examined at a range of 125–250 W while all other experimental variables were kept constant. Fig. 1F depicted that the TFC exhibited a progressive increase as ultrasonic power increased, reaching optimal value at 200 W, but subsequently decreased with elevated ultrasonic power. Within an appropriate range, enhanced ultrasonic power was conducive to destroying cells and promoting the release of intracellular components [31]. Meanwhile, the mechanical effects generated by ultrasound enhanced solvent diffusion, facilitating mass transfer processes [32]. Once the ultrasonic power exceeded the optimal level, the intense cavitation effect could generate localized high temperatures, pressures, breaking chemical bonds of certain flavonoids, resulting in structural degradation and yield reduction of flavonoids [33]. To maximize the total flavonoid yield while saving energy, 200 W was determined as the ideal condition for subsequent extraction process after a comprehensive investigation.

#### 3.2.5 Ethanol volume fraction

As a green solvent, ethanol is widely used in the extraction of flavonoids [34]. Ethanol volume fraction is a pivotal variable that directly modulates solvent polarity and influences the dissolution behavior of bioactive constituents. As shown in Fig. 1G, the TFC obtained from CM exhibited a continuous upward trend as ethanol volume fraction increased from 30 % to 80 %, reaching its optimal level (20.30 ± 0.25 mg/g) at an ethanol volume fraction of 70 %. When the ethanol concentration ranged between 70 and 80 %, the TFC exhibited a downward trend. This trend could be attributed to two factors: first, a higher ethanol volume fraction promoted the dissolution and extraction of flavonoids owing to ethanol’s favorable solubility and strong cellular permeability [10]. Second, an appropriate ethanol volume fraction provided a polarity similar to that of flavonoid compounds, allowing stronger interactions and improved solubility [35]. However, excessively high ethanol volume fraction adversely affected the extraction by inducing cellular dehydration, promoting structural collapse of plant tissues, and denaturing cell wall-associated proteins [36]. Hence, an ethanol volume fraction of 70 % was selected as the most suitable condition for efficient flavonoid extraction from CM.

### 3.3 PBD for screening significant factors

PBD was utilized to optimize five pivotal parameters influencing the extraction of flavonoids from CM, including solid-to-liquid ratio (A), ultrasonic time (B), ultrasonic temperature (C), ultrasonic power (D), and ethanol volume fraction (E). The experimental design matrix, along with the corresponding TFC as the response variable, was summarized in Table S6. The first-order polynomial equation for TFC is expressed as *Eq.* (5).

(5) Y=18.95+1.2A+0.1457B-0.7075C+0.0810D+0.3185E

According to the ANOVA results, the *F* value of 51.02 (*P* < 0.0001) demonstrated the statistical significance of PBD model. The adjusted model’s coefficient of determination (*R^2^*) was 0.9579, suggesting that the model could illustrate 95.79 % of the response variability, which further verified the reliability of this model (Table 1). In general, a confidence level of the parameter exceeded 95 % (*P* < 0.05) indicated that the parameter was significantly related to the response value [37]. Among the tested factors, solid-to-liquid ratio (A), ultrasonic temperature (C), and ethanol volume fraction (E) were considered to significantly affect the extraction of flavonoid compounds from CM (*P* < 0.05), whereas other parameters had no significant impacts.

**Table 1:** ANOVA analysis of Plackett-Burman Design.

**Source**	**Sum of squares**	**Degrees of freedom**	**Mean sum of squares**	***F* value**	***P* value**
Model	24.73	5	4.95	51.02	< 0.0001****
A	17.17	1	17.17	177.16	< 0.0001****
B	0.2546	1	0.2546	2.63	0.1562
C	6.01	1	6.01	61.97	0.0002***
D	0.0787	1	0.0787	0.8117	0.4023
E	1.22	1	1.22	12.56	0.0122*
Residual	0.5816	6	0.0969		
Cor Total	25.31	11			
Std. Dev.	0.3113			*R^2^*	0.977
Mean	18.95			Adjusted *R^2^*	0.9579
C.V. %	1.64			Predicted *R^2^*	0.9081
				Adeq Precision	20.1884

****P < 0.0001, ***P < 0.001, *P < 0.05.

**Fig. 2:**
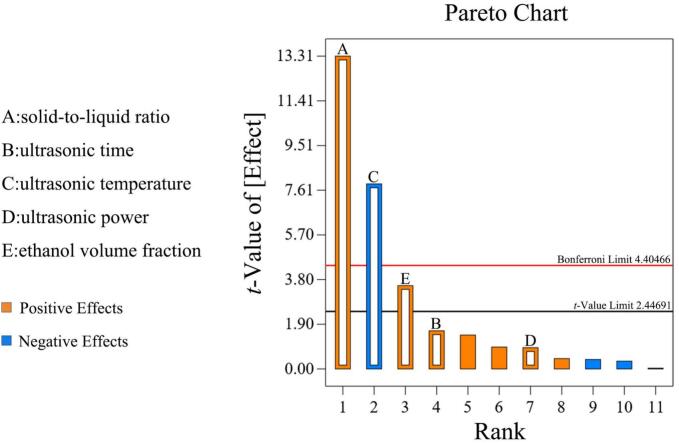
Pareto plot illustrating the relative influence of five experimental factors on TFC. Factors exhibiting *t*-values exceeding the threshold of statistical significance (2.45) were considered to have a significant effect.

To visualize the relative contributions of experimental variables on the extraction of TFC from CM, a Pareto chart was conducted and presented graphically. The standardized effects of each factor were compared to the Bonferroni (4.40) and the critical *t*-value (2.45) to evaluate the significance of the results [38]. As shown in Fig. 2, solid-to-liquid ratio (A), ultrasonic time (B), ultrasonic power (D), and ethanol volume fraction (E) positively affected the responses, while ultrasonic temperature (C) negatively contributed to the flavonoid yield. Among these, the determined significant parameters were solid-to-liquid ratio (A), ultrasonic temperature (C), and ethanol volume fraction (E), with effect values exceeding the t-value threshold, consistent with the ANOVA results in Table 1. Consequently, solid-to-liquid ratio (A), ultrasonic temperature (C), and ethanol volume fraction (E) were further optimized in BBD test.

### 3.4 RSM analysis for optimizing flavonoid yield of CM

#### 3.4.1 Model fitting

On the basis of preliminary optimization using PBD, RSM analysis was utilized to further optimize the extraction parameters of TFC in CM extracts. In the RSM experiment, three key variables, including solid-to-liquid ratio (A), ultrasonic temperature (C), and ethanol volume fraction (E), were each tested at three levels. According to BBD principles, 17 experimental runs were generated utilizing Design-Expert 13, comprising 12 interactive experiments and 5 central repeated experiments, and the results were displayed in Table S7. In addition, a multivariate regression approach was applied to predict complex relationships between the three variables and the total flavonoid content (*Y*), while the corresponding second-order polynomial model was as *Eq.* (6):

(6) $$Y=20.74+0.6146A-0.4384C-0.6933E+0.2776AC+0.4901AE-0.3598CE-1.05A^{2}-1.05C^{2}-3.04E^{2}$$

As shown in Table 2, the statistical adequacy and predictive robustness of the regression model were examined using the *F*-statistic, *P*-values, and the coefficients of determination. ANOVA analysis revealed a markedly high model *F* statistic (170.99) with a *P* value below 0.0001, while the lack-of-fit test yielded a non-significant result (*F* = 0.1636, *P* > 0.05), indicating that the model was statistically valid and reliable for optimization analysis. Additionally, the model’s *R^2^* was 0.9955, indicating a strong linear relationship between the independent and dependent variables, implying minimal deviation between predicted and experimental measurements. The adjusted *R^2^* value of 0.9896 also confirmed the strong predictive power of RSM model. Moreover, the low coefficient of variation (C.V. % = 1.1) highlighted the reliability and repeatability of the experimental procedure. Meanwhile, the signal-to-noise ratio (Adeq precision = 38.1665) was much greater than 4, which further supported the reliability of the model [39]. In regression analysis, the *P*-value was utilized to assess both the significance of each variable and the interplay among them. All the terms, including individual factors (A, C, E), quadratic terms (A^2^, C^2^, E^2^), and the interaction between the factors (AC, AE, CE), exhibited significant effects. Based on the *F*-value, the influence of the three parameters on *Y* followed the order: E (ethanol volume fraction) > A (solid-to-liquid ratio) > C (ultrasonic temperature).

**Table 2:** ANOVA summary for the regression model of BBD.

**Source**	**Sum of squares**	**Degrees of freedom**	**Mean sum of squares**	***F-*value**	***P-*value**
Model	62.34	9	6.93	170.99	<0.0001****
A	3.02	1	3.02	74.59	<0.0001****
C	1.54	1	1.54	37.95	0.0005***
E	3.85	1	3.85	94.93	<0.0001****
AC	0.3083	1	0.3083	7.61	0.0282*
AE	0.9607	1	0.9607	23.72	0.0018**
CE	0.5178	1	0.5178	12.78	0.009**
A^2^	4.64	1	4.64	114.53	<0.0001****
C^2^	4.68	1	4.68	115.45	<0.0001****
E^2^	39.02	1	39.02	963.08	<0.0001****
Residual	0.2836	7	0.0405		
Lack of Fit	0.031	3	0.0103	0.1636	0.9156
Pure Error	0.2526	4	0.0631		
Cor Total	62.63	16			
Std. Dev.	0.2013			*R^2^*	0.9955
Mean	18.32			Adjusted *R^2^*	0.9896
C.V. %	1.1			Predicted *R^2^*	0.9858
			Adeq Precision		38.1665

****P < 0.0001, ***P < 0.001, **P < 0.01, *P < 0.05.

#### 3.4.2 RSM analysis

**Fig. 3:**
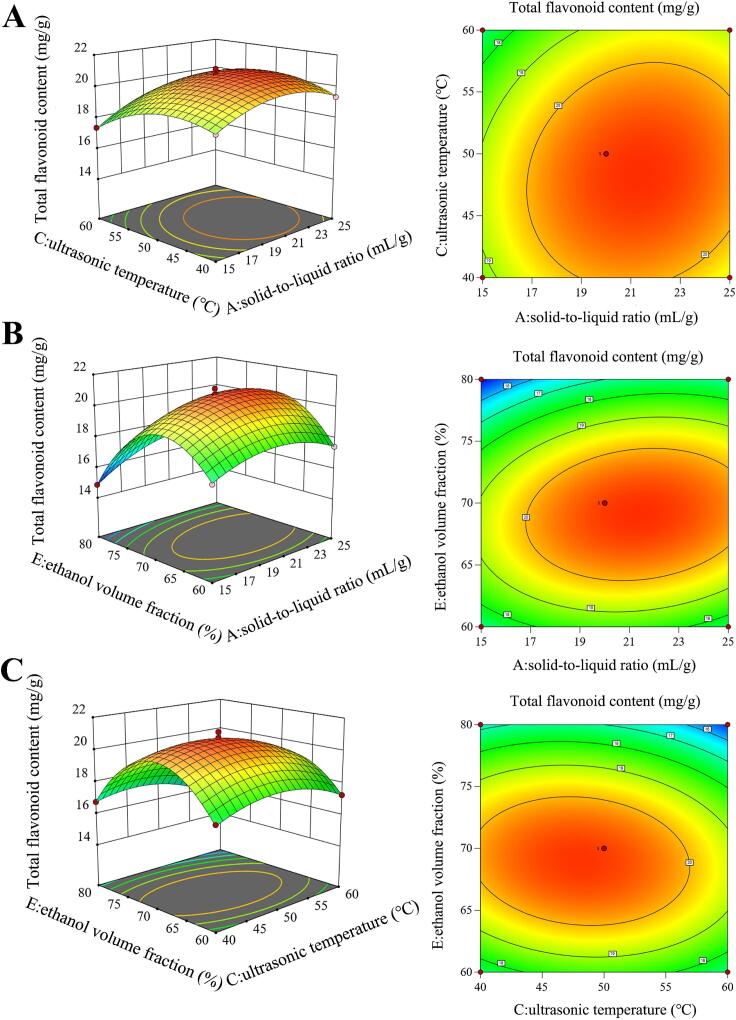
The interactive relationship between critical variables. Three-dimensional response surface and the two-dimensional contour plots between A and C (A), A and E (B), and C and E (C).

To intuitively elucidate the interactions between extraction parameters, three-dimensional response surface diagrams and two-dimensional contour plots were employed. Fig. 3A showed the interaction between solid-to-liquid ratio and ultrasonic temperature. The relatively gentle response surface and an elliptical contour line indicated a non-significant interaction between the two factors during the extraction process. On the contrary, as depicted in Fig. 3B, the yield of flavonoid showed a steep increase with the elevating ethanol concentration under fixed solid-to-liquid ratio. The corresponding two-dimensional contour plots were elliptical and dense, suggesting a significant interaction between ethanol volume fraction and solid-to-liquid ratio in extracting flavonoid from CM. Similarly, the interaction term of ultrasonic temperature and ethanol volume fraction showed a steep surface and dense elliptical contour plot, indicating a significant interaction between two factors (Fig. 3C). These findings were in line with the ANOVA analysis summary listed in Table 2.

### 3.5 BP-GA analysis of conditions for optimizing CM flavonoid content

**Fig. 4:**
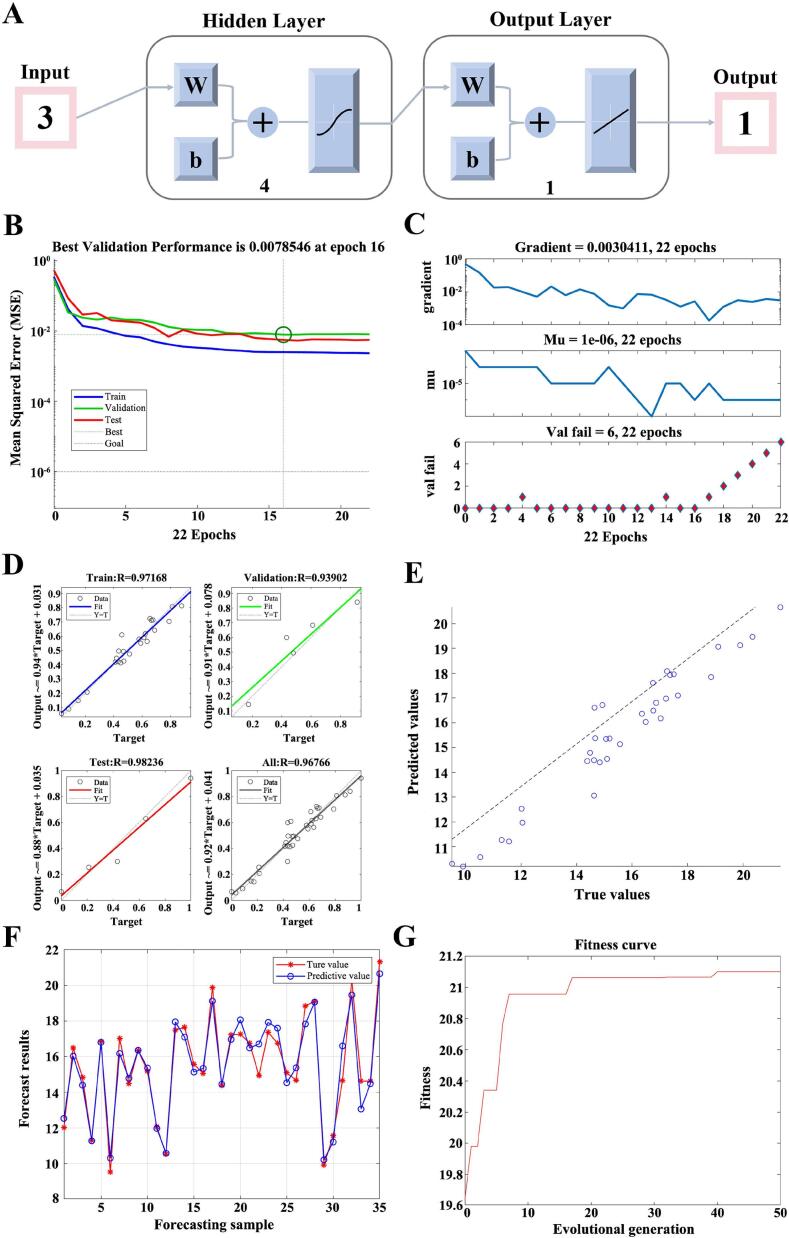
Outcomes of the BP-GA optimization procedure. (A) Architecture of the BP-GA neural network. (B) Best validation performance, (C) Training state, (D) Regression analysis, (E, F) The comparison of actual values and predicted values, (G) GA model optimization.

As a nonlinear mathematical model inspired by biological neural networks, BP-GA has shown strong capability in handling the prediction and optimization problems, particularly in extraction-related applications of traditional herbal materials [40]. In the present study, BP-GA was employed to establish a quantitative relationship between three key processing parameters and the TFC. Briefly, a BP neural network was constructed with 3 input layers (ethanol volume fraction, solid-to-liquid ratio, and ultrasonic temperature), 1 output layer (the total flavonoid content) and 4 hidden layer neurons (Fig. 4A). As shown in Fig. 4B, the network reached optimal training performance with a minimum mean squared error (0.0078546) after 16 epochs, indicating that the model training reached the best state at 16 epochs. Fig. 4C demonstrated that the gradient value was 0.0030411, mu was 10^-6^, and val fail was 6, suggesting that the neural network model was well trained. The R of correlation coefficients for the train, validation, test and overall regression curve were 0.97168, 0.93902, 0.98236 and 0.96766, respectively, implying a strong correlation between actual and predicted values (Fig. 4D). Additionally, a series of parameters were calculated to evaluate the fit quality, including MAE (0.54359), MSE (0.014808), RMSE (0.71992) and *R^2^* (0.93604). The fitting situation between the actual value and the predicted value was shown in Fig. 4E and F. These results further confirmed that the BP-GA neural network was successfully established, which could be used to optimize the extraction process.

### 3.6 Experimental verification and comparison of RSM and BP-GA optimization results

The optimal parameters identified by RSM were a solid-to-liquid ratio of 1: 21.26, an ultrasonic temperature of 48.40 ℃, and an ethanol volume fraction of 69.16 %. In comparison, the optimized process of BP-GA was shown in Fig. 4G, and the conditions were as follows: solid-to-liquid ratio was 1: 24.26, ultrasonic temperature was 54.88 ℃, and ethanol volume fraction was 67.11 %. To verify the reliability of predictive results, three repeated experiments were conducted according to the optimized conditions obtained by RSM and BP-GA (Table 3). Results showed that the BP-GA model exhibited superior predictive performance in optimizing TFC, with a relative error of 0.22 % compared to 1.55 % for RSM. In addition, as displayed in Table 3, optimized TFC by BP-GA was 21.18 ± 0.94 mg/g, exceeding that obtained through RSM (20.56 ± 0.09 mg/g). These results indicated that the BP-GA model outperformed RSM in terms of both lower prediction error and higher flavonoid yield. Notably, under the optimized conditions, the TFC obtained by UAEE was higher than those reported for CM [41], [42], suggesting that the UAEE method combined with BP-GA optimization effectively promoted the extraction of flavonoids from CM.

**Table 3:** Comparison of optimization results.

**Optimistic method**	**solid-to-liquid ratio** **(g/mL)**	**ultrasonic temperature** **(℃)**	**ethanol volume fraction (%)**	**Predicted value** **(mg/g)**	**Actual value** **(mean ± SD, mg/g)**	**Relative error** **(%)**
RSM	1: 21.26	48.40	69.16	20.88	20.56 ± 0.09	1.55
BP-GA	1: 24.26	54.88	67.11	21.14	21.18 ± 0.94	0.22

### 3.7 Mechanism of UAEE process

#### 3.7.1 Cell wall compositional changes of CM extracts

**Fig. 5:**
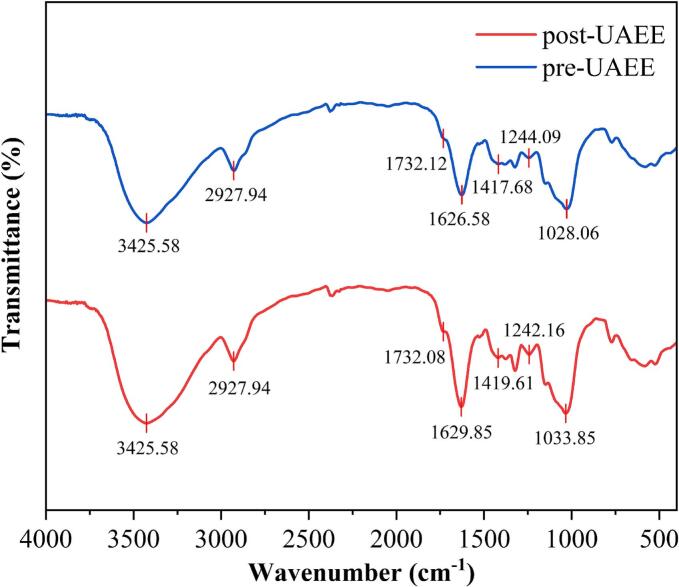
FT-IR spectroscopy alteration of cell wall constituents in CM extracts.

FT-IR spectroscopy was utilized to examine changes in cell wall constituents following UAEE (Fig. 5). The band at 3425 cm^−1^ corresponded to O-H stretching vibrations originating from cellulose and hemicellulose, along with intramolecular hydrogen bonding [43]. The peak at 2927 cm^−1^ was attributed to the characteristic of amorphous cellulose generated by C-H stretching vibrations in methyl, methylene, and methine groups [44]. A signal centered around 1732 cm^−1^ was attributed to the C=O stretching vibration from carboxyl groups in hemicellulose [45], its intensity altered after UAEE, suggesting possible structural alterations in hemicellulose. The band located at 1629 cm^−1^ is assigned to asymmetric C=C stretching vibrations. Meanwhile, at 1419 cm^−1^, the absorption corresponded to bending vibrations of –CH_2_ and –CH_3_ groups attached to carboxyl and ester functionalities found in lignin and cellulose [43]. Furthermore, the signal located at approximately 1244 cm^−1^ is associated with terpenoid skeleton vibrations, associated with C-O stretching between hemicellulose and lignin. Whereas the absorption at 1028 cm^−1^ was related to C-O stretching of the pyranose ring in cellulose and hemicellulose [46]. After UAEE, the increased intensity of these characteristic bands indicated greater exposure of functional groups in cellulose, hemicellulose, and lignin. This observation suggested modifications in the crystalline structure of cellulose and a breakdown of the cell wall matrix.

#### 3.7.2 Surface morphology analysis of CM samples

**Fig. 6:**
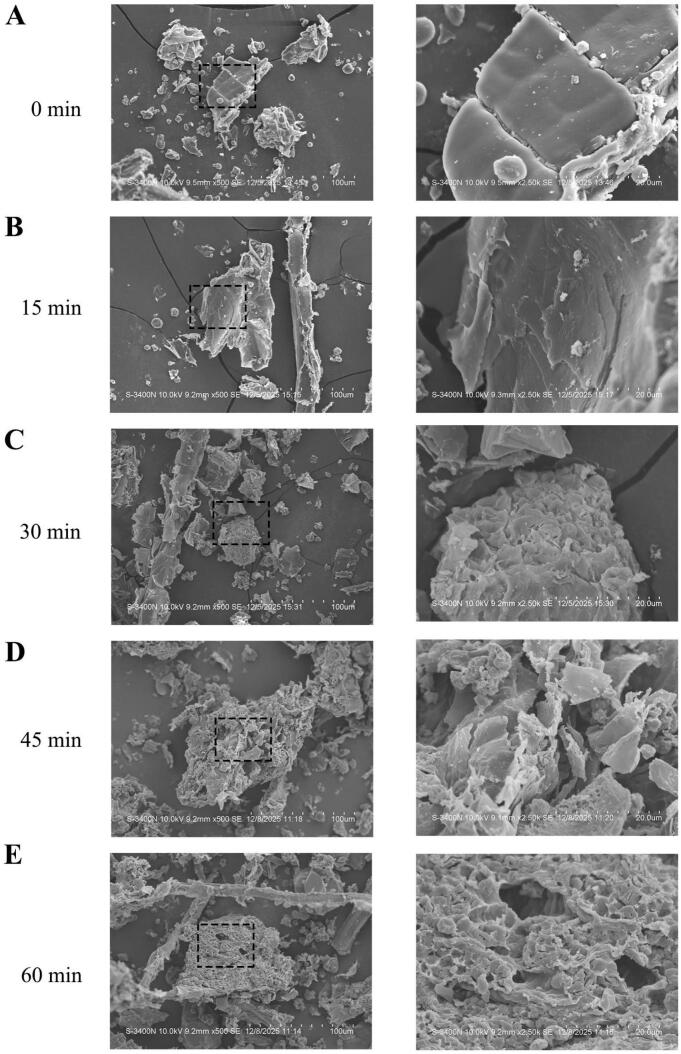
SEM images of CM extracts at different treated time. (A) 0 min, (B) 15 min, (C) 30 min, (D) 45 min, (E) 60 min.

SEM was employed to examine the microstructural characteristics of CM samples before and after UAEE treatment at magnifications of 500 × and 2500 × . As displayed in Fig. 6A, the untreated sample surface exhibited compact and continuous, with a well-preserved microstructural framework before sonication. After ultrasonic exposure, surface morphology changed progressively with increasing exposure time (Fig. 6B-E). After 15 min, slight surface irregularities and initial fissures began to appear (Fig. 6B). As the treatment time increased to 30 and 45 min, the original framework was progressively disrupted, accompanied by apparent fragmentation and emerging pores (Fig. 6C-D). At 60 min, the surface exhibited extensive disruption and a porous architecture (Fig. 6E). This morphological evolution reflected the cumulative impact of ultrasonic cavitation and mechanical forces. Repeated collapse of cavitation bubbles generated by ultrasound promoted surface erosion and internal collapse, resulting in pronounced porosity and fragmentation. Further extended processing time led to honeycomb-like architecture, indicating substantial disintegration of the internal matrix and facilitating solvent penetration and mass transfer.

#### 3.7.3 MDS of CM extraction process

**Fig. 7:**
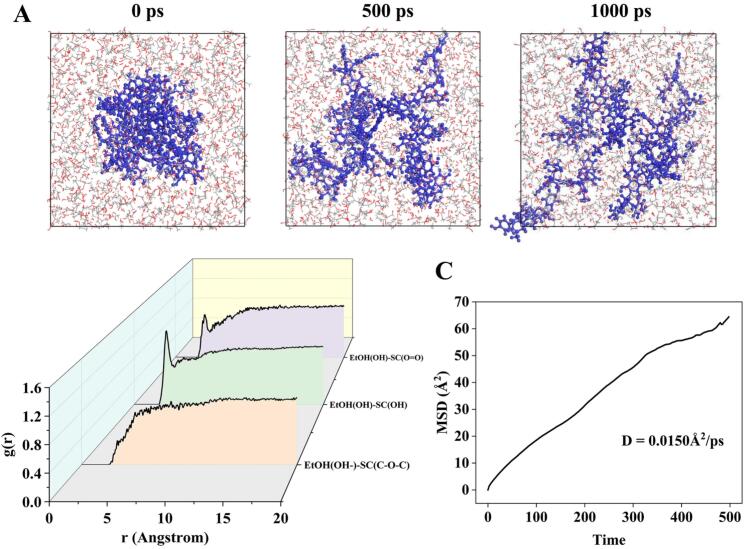
The MDS of extracting TFC from CM. (A) The distribution of sanggenon C in 67 % ethanol–water system. (B) The RDF analysis of between sanggenon C and ethanol. (C) The MSD analysis of sanggenon C in 67 % ethanol–water system.

To evaluate the dynamic spatial distribution characteristics of TFC during the extracting process, sanggenon C was selected as a representative compound to investigate its distribution and interaction in ethanol–water system by simulating the mechanical effect of ultrasound through an external force field. Results showed that the small molecules were aggregated before the simulation started, but became progressively and uniformly dispersed in solvent system as the simulation time increased (Fig. 7A). RDF analysis was performed to characterize the spatial density distribution of particles around small molecules. Peaks in the range of 1.5–3.5 Å represented hydrogen bonding interactions, and van der Waals forces were generally identified by peaks in 3.5–5.0 Å [47]. Fig. 7B displayed the hydrogen bonding interactions between the hydroxyl and carbonyl in sanggenon C and hydroxyl in ethanol molecules, which were confirmed by the peaks at 3.27 Å and 3.41 Å, respectively. Moreover, the weak peak in 3.5–5.0 Å implied the presence of van der Waals interactions, suggesting that solvent–solute interactions promoted dissolution through hydrogen bonding and van der Waals interaction forces. MSD results demonstrated that the MSD value of sanggenon C in ethanol–water system was 64.4087 Å^2^ and the diffusion coefficients were 0.0150 Å^2^/ps (Fig. 7C), indicating powerful solute–solvent interaction and diffusion characteristics. These results indicated that the diffusion of small molecules and the solvent mass transfer were enhanced by ultrasonic treatment, thus facilitating the TFC extraction.

### 3.8 Qualitative analysis of flavonoid compounds in CM

**Fig. 8:**
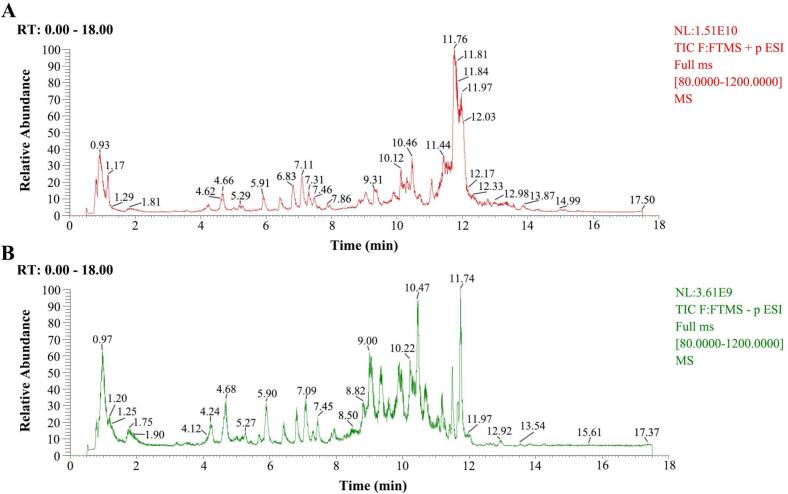
Total ion chromatograms of CM extracts in positive (A) and negative (B) mode.

**Fig. 9:**
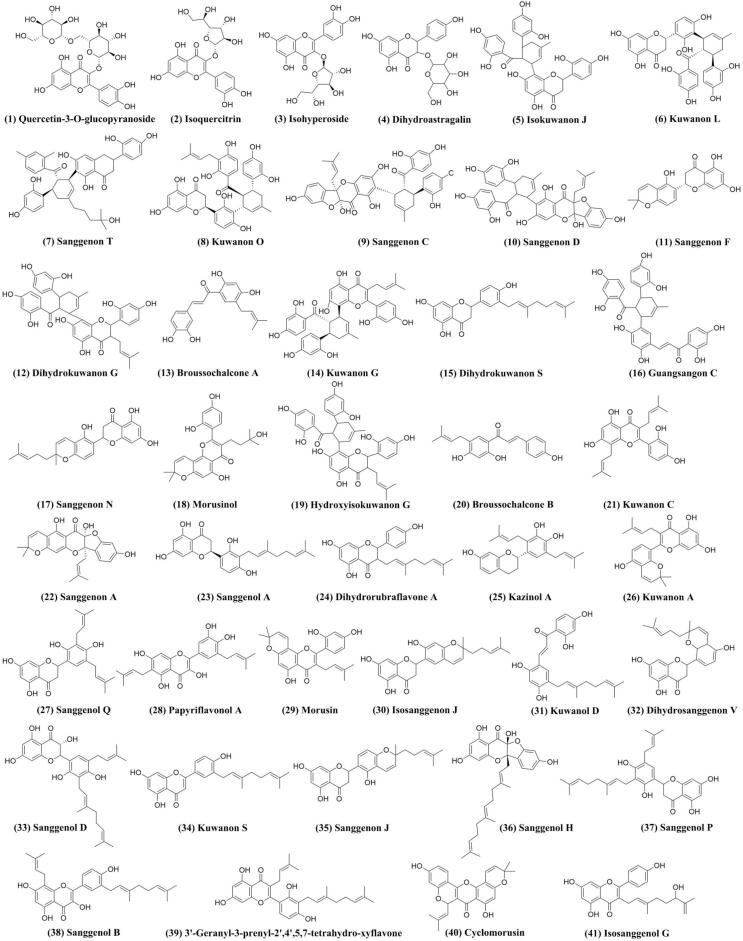
Structures of identified flavonoid compounds in CM extracts.

UHPLC-HRMS was utilized to characterize the flavonoid composition of CM extracts and the chromatograms were shown in Fig. 8. The components were identified based on relevant literature by comparing the mass-to-charge ratio and MS/MS fragmentation patterns. As summarized in Table S8, a total of 41 flavonoids were characterized, including 40 in negative ion modes and 1 in positive ion modes, indicating that flavonoids constituted the major class of identified compounds. The structures of flavonoids were displayed in Fig. 9, consistent with previous studies that CM is an exceptionally rich source of flavonoids. Nevertheless, the diversity of flavonoids differed from that reported in other studies, which may be attributed to different extraction conditions, indicating that parameter optimization not only affected the TFC, but also influenced the compositional profile of flavonoids in CM extracts [48]. These findings contributed to developing more efficient extraction strategies and promoting the application of flavonoids from CM.

### 3.9 Antioxidant activity of CM extracts in vitro

Flavonoids, a large class of polyphenolic compounds, exhibit strong antioxidant activity due to their unique chemical structure with multiple hydroxyl groups, allowing them to neutralize harmful free radicals by donating electrons [49]. Hence, this study investigated the antioxidant activity of CM extracts by assessing radical scavenging ability and FRAP.

#### 3.9.1 ABTS^+^ radical scavenging ability

**Fig. 10:**
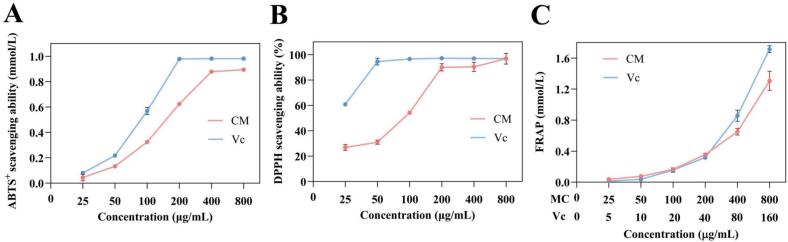
*In vitro* evaluation of the antioxidant properties of CM extracts. (A) ABTS^+^ scavenging ability. (B) DPPH scavenging ability. (C) FRAP. n = 3, data represent mean ± SD.

To quantify antioxidant potential, the capacity to scavenge ABTS^+^ radicals was measured as a representative index [50]. Fig. 10A illustrated that the ABTS^+^ radical scavenging ability showed a clear concentration-dependent enhancement within 25–––400 μg/mL, and the rising trend leveled off beyond 400 μg/mL. The ABTS^+^ radical scavenging ability reached 0.8940 ± 0.0010 mmol/L at 800 μg/mL, which was close to that of the ascorbic acid (Vc) group (0.9817 ± 0.0010 mmol/L), indicating that the hydroxyl groups in CM extracts could effectively scavenge ABTS^+^ radicals by donating electrons.

#### 3.9.2 DPPH radical scavenging ability

The DPPH radical, a stable nitrogen-centered free radical, is widely applied in evaluating antioxidant properties in vitro [51]. As shown in Fig. 10B, the DPPH radical scavenging activity was positively associated with the concentration of CM extracts ranging from 25 to 200 μg/mL. At concentrations above 200 μg/mL, the DPPH radical scavenging ability gradually plateaued and was maintained at a level comparable to Vc group, indicating a strong DPPH radical scavenging capacity of the CM extracts.

#### 3.9.3 FRAP assay

Ferric ions (Fe^3+^) can be reduced by antioxidants to ferrous ions (Fe^2+^), and the resulting Fe^2+^ concentration was directly proportional to the antioxidant capacity in the sample [52]. As the concentration of CM extracts increased from 25 to 800 μg/mL, the FRAP value rose from 0.0361 ± 0.0017 mmol/L to 1.3074 ± 0.1234 mmol/L, displaying same trend and value as Vc (Fig. 10C). This phenomenon was aligned with previous report proposed by Li et al. [34], the ferric reducing power of CM extracts was largely derived from the presence of adjacent hydroxyl structures situated on the phenolic rings in flavonoid compounds. Overall, CM extracts obtained by UAEE exhibited powerful antioxidant activity and showed potential for development as effective antioxidants.

### 3.10 Anti-cancer activity of CM extracts in vitro

**Fig. 11:**
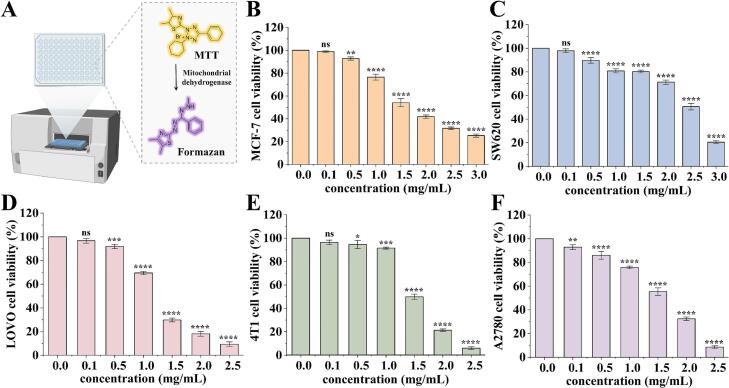
Anti-cancer activity of CM extracts. (A) The principle of MTT assay. Cell viability of MCF-7 (B), SW620 (C), LOVO (D), 4 T1 (E), and A2780 (F) cells after 24 h treatment of CM extracts. n = 3, data represent mean ± SD. *P < 0.05, **P < 0.01, ***P < 0.001, ****P < 0.0001, ns, not significant, vs the normal group.

The in vitro antiproliferative potential of CM extracts was evaluated using MTT assays, which assessed their effects on proliferation of SW620, 4 T1, A2780, LOVO, and MCF-7 cells (Fig. 11A). As illustrated in Fig. 11B, the CM extracts inhibited MCF-7 cell proliferation as the concentration progressed from 0.1 to 3.0 mg/mL, with cell viability decreasing from 98.81 ± 0.67 % to 25.29 ± 1.33 % and an IC_50_ value of 1.704 mg/mL. Similarly, the cell viability of SW620 was inhibited by CM extracts and fell below 50 % at 2.5 mg/mL (Fig. 11C). Moreover, we also observed that the anti-proliferation activity against LOVO, 4 T1 and A2780 cells increased as the concentration elevated from 0.1 mg/mL to 2.5 mg/mL, with IC_50_ value of 1.225, 1.517, and 1.501 mg/mL, respectively (Fig. 11D-F). Altogether, CM extracts exhibited concentration-dependent antiproliferative effects against multiple cancer cell lines, which is consistent with previous reports on the bioactivity of flavonoids [49], suggesting their potential anticancer activity.

## 4 Conclusion

This research firstly presents an innovative means for extracting flavonoids from CM by UAEE, combined with a parameter optimization integrating PBD, RSM and BP-GA. The utilization of UAEE significantly enhanced the yield of flavonoids in CM extracts after parameter optimization, which may be attributed to enhanced solvent accessibility, intermolecular interactions and structural disruption caused by ultrasound. Bioactivity assessment demonstrated that CM extracts possessed powerful antioxidant properties and could inhibit the proliferation of various cancer cell lines at elevated concentrations, which may be attributed to abundant flavonoid compounds. These findings offer a theoretical underpinning for the potential application of CM flavonoids in the medical and healthcare fields and provide an efficient protocol for the extraction and optimization of flavonoids from natural plants.

## 5 Consent to participate

Informed consent was obtained from all participants.

## 6 Consent for publication

Not applicable.

## CRediT authorship contribution statement

**Haixia Che:** Writing – review & editing, Writing – original draft, Data curation. **Jiawen Li:** Validation, Methodology, Data curation. **Huiru Fu:** Investigation. **Yue Hu:** Investigation. **Di Gao:** Investigation. **Zejun Li:** Validation. **Qiao Li:** Validation. **Yu Xiao:** Methodology. **Yue Zhou:** Validation. **Mingming Lian:** Validation, Methodology. **Qian Li:** Validation.

## Ethics approval

Not applicable.

## Declaration of competing interest

The authors declare that they have no known competing financial interests or personal relationships that could have appeared to influence the work reported in this paper.

## Data Availability

The data that support the findings of this study are available from the corresponding author upon reasonable request.

## Appendix A Supplementary data

Supplementary Data 1 Supplementary material including experimental design, UHPLC-HRMS analytical conditions, and compound identification data for CM extracts.
