# Supplementary material for: Integration of ANN-GA optimization and multi-scale mechanistic analysis for ultrasound-assisted enzymatic extraction of flavonoids from Cortex Mori
Source: Ultrason Sonochem. 2026 May 24;130:107906. doi: 10.1016/j.ultsonch.2026.107906 (PMC13234740; doi:10.1016/j.ultsonch.2026.107906)
Supplement: Supplementary Data 1 — Supplementary material including experimental design, UHPLC-HRMS analytical conditions, and compound identification data for CM extracts. [file mmc1.docx]

**Supplementary materials**

**Table. S1 Materials and regents**

| **Materials** | **Manufactor** |
| --- | --- |
| Rutin (purity ≥ 95%) | China Biological Products Certification Institute |
| 2,2-Diphenyl-1-picrylhydrazyl (DPPH) | Macklin |
| Methanol (HPLC-grade) | MREDA |
| Formic acid | Aladdin |
| Dimethyl sulfoxide (DMSO) | Aladdin |
| 3-(4,5-dimethylthiazol-2-yl)-2,5-diphenyltetrazolium bromide (MTT) | Biosharp |
| 2,2-Azino-bis-(3-ethylbenzothiazoline-6-sulfonic acid) (ABTS) assay kit | Nanjing Jiancheng Bioengineering Institute |
| Ferric reducing antioxidant power (FRAP) assay kit | Nanjing Jiancheng Bioengineering Institute |
| Dulbecco’s modified eagle medium (DMEM) | Cytiva |
| Fetal Bovine Serum (FBS) | Zhejiang Tianhang Biotechnology Co., Ltd. |

**Table. S2 Factors and levels of Plackett-Burman design for TFC extraction from CM.**

| Factors | Levels | |
| --- | --- | --- |
|  | -1 | 1 |
| A (liquid-to-solid ratio/mL/g) | 15: 1 | 25: 1 |
| B (ultrasonic time/min) | 45 | 75 |
| C (ultrasonic temperature/℃) | 40 | 60 |
| D (ultrasonic power/W) | 175 | 225 |
| E (ethanol volume fraction/%) | 60 | 80 |

**Table S3. Variables and levels for Box-Behnken Design.**

| Factors | Levels | | |
| --- | --- | --- | --- |
|  | -1 | 0 | 1 |
| A (solid-to-liquid ratio/g/mL) | 15: 1 | 20: 1 | 25: 1 |
| C (ultrasonic temperature/℃) | 40 | 50 | 60 |
| E (ethanol volume fraction/%) | 60 | 70 | 80 |

**Table S4.UHPLC and MS parameters for detection of CM extracts.**

| **UPLC/MS conditions** | **Parameters** |
| --- | --- |
| Chromatographic column | Hypersil GOLD VANQUISH column (1.9 μm, 2.1×100 mm) |
| Mobile phase | (A) 0.1% formic acid in water (B) methanol |
| Flow rate | 0.3 mL/min |
| Injection volume | 3 μL |
| Column temperature | 35 °C |
| Ion Source Type | heated electrospray ionization (HESI) source |
| ESI modes | Positive (+), negative (-) |
| Orbitrap Resolution | 60000 |
| Scan Range | m/z 80 ~ 1200 |
| RF lens (%) | 70 |
| Capillary voltage | 3400 V (+), 2300 V (-) |
| Sheath gas | 30 |
| Aux Gas | 10 |
| Sweep Gas | 0 |
| Ion Transfer Tube Temp（℃） | 320 ℃ |
| Vaporizer Temp（℃） | 320 ℃ |

**Table S5. The gradient elution conditions of the UHPLC system**

| **T (min)** | **A (%)** | **B (%)** |
| --- | --- | --- |
| 0 | 98 | 2 |
| 0.5 | 98 | 2 |
| 10 | 2 | 98 |
| 16 | 2 | 98 |
| 17.1 | 98 | 2 |
| 18.0 | 98 | 2 |

**Table S6.** **Experimental design and response value of Plackett-Burman Design.**

| Run order | Factor | | | | | Total flavonoid content (mg/g) |
| --- | --- | --- | --- | --- | --- | --- |
|  | A (g/mL) | B (min) | C (℃) | D (W) | E (%) |  |
| 1 | 15 | 75 | 40 | 225 | 80 | 18.6944402 |
| 2 | 25 | 45 | 60 | 225 | 80 | 19.62758996 |
| 3 | 25 | 45 | 40 | 175 | 80 | 21.24232998 |
| 4 | 25 | 75 | 40 | 225 | 80 | 21.62476841 |
| 5 | 15 | 75 | 60 | 225 | 60 | 17.41114681 |
| 6 | 15 | 45 | 40 | 225 | 60 | 17.91256608 |
| 7 | 15 | 45 | 60 | 175 | 80 | 17.04570564 |
| 8 | 25 | 45 | 60 | 225 | 60 | 18.93353503 |
| 9 | 25 | 75 | 60 | 175 | 60 | 19.06101451 |
| 10 | 15 | 45 | 40 | 175 | 60 | 18.08253871 |
| 11 | 15 | 75 | 60 | 175 | 80 | 17.39414954 |
| 12 | 25 | 75 | 40 | 175 | 60 | 20.4066312 |

**Table S7. Experimental design and response value of Box-Behnken.**

| Run order | Factor | | | Response |
| --- | --- | --- | --- | --- |
|  | A (g/mL) | C (℃) | E (%) | Total flavonoid content (mg/g) |
| 1 | 20 | 50 | 70 | 20.49530026 |
| 2 | 15 | 60 | 70 | 17.36865365 |
| 3 | 20 | 60 | 80 | 15.06750746 |
| 4 | 20 | 50 | 70 | 20.76725647 |
| 5 | 20 | 40 | 80 | 16.74457079 |
| 6 | 25 | 60 | 70 | 19.10350767 |
| 7 | 20 | 60 | 60 | 17.25448869 |
| 8 | 15 | 50 | 80 | 14.86155729 |
| 9 | 20 | 50 | 70 | 20.75592496 |
| 10 | 15 | 50 | 60 | 17.14768922 |
| 11 | 20 | 50 | 70 | 21.12986476 |
| 12 | 25 | 40 | 70 | 19.34430223 |
| 13 | 25 | 50 | 60 | 17.44627448 |
| 14 | 25 | 50 | 80 | 17.1204936 |
| 15 | 20 | 50 | 70 | 20.54062629 |
| 16 | 15 | 40 | 70 | 18.71993609 |
| 17 | 20 | 40 | 60 | 17.49245038 |

**Table S8. The compounds identified in CM extracts using UHPLC-HRMS.**

| **No.** | **RT**  **(min)** | **Compounds** | **Molecular**  **formula** | **Ion mode** | **Molecular**  **weigh** | **Measured**  **(m/z)** | **Fragment irons** | **Ref.** |
| --- | --- | --- | --- | --- | --- | --- | --- | --- |
| 1 | 4.19 | Quercetin-3-O-glucopyranoside | C_21_H_22_O_12_ | [M-H]- | 466.1116 | 465.1058 | 437.1081, 303.0511, 125.0243 | [1] |
| 2 | 5.35 | Isoquercitrin | C_21_H_20_O_12_ | [M-H]- | 464.0954 | 463.0876 | 301.0350, 273.0398 | [2] |
| 3 | 5.49 | Isohyperoside | C_21_H_20_O_12_ | [M-H]- | 464.0960 | 463.0880 | 301.0350, 151.0034 | [1] |
| 4 | 6.35 | Dihydroastragalin | C_21_H_22_O_11_ | [M-H]- | 450.1156 | 449.1075 | 287.0568, 151.0038, 125.0243 | [1] |
| 5 | 8.10 | Isokuwanon J | C_35_H_30_O_11_ | [M-H]- | 626.1793 | 625.1705 | 109.0294 | [1] |
| 6 | 8.15 | Kuwanon L | C_35_H_30_O_11_ | [M-H]- | 626.1788 | 625.1707 | 607.1629, 515.1381 | [2] |
| 7 | 8.58 | Sanggenon T | C_40_H_40_O_12_ | [M-H]- | 712.2519 | 711.2402 | 601.2057, 549.2126, 439.1750 | [2] |
| 8 | 8.61 | Kuwanon O | C_40_H_38_O_11_ | [M-H]- | 694.2420 | 693.2335 | 109.0294, 125.0243, 161.0243, 135.0450 | [3] |
| 9 | 9.04 | Sanggenon C | C_40_H_36_O_12_ | [M-H]- | 708.2212 | 707.2138 | 109.0294, 125.0244, 151.0036, 191.0713, 217.0506, 243.0661, 435.1442, 501.1185, 638.1447 | [3] |
| 10 | 9.09 | Sanggenon D | C_40_H_36_O_12_ | [M-H]- | 708.2206 | 707.2155 | 353.1034, 638.1424, 597.1763 | [2] |
| 11 | 9.29 | Sanggenon F | C_20_H_18_O_6_ | [M-H]- | 354.1108 | 353.1020 | 177.0192, 125.0243 | [1] |
| 12 | 9.31 | Dihydrokuwanon G | C_40_H_38_O_11_ | [M-H]- | 694.2419 | 693.2300 | 567.2052, 389.1021, 125.0243 | [1] |
| 13 | 9.33 | Broussochalcone A | C_20_H_20_O_5_ | [M-H]- | 340.1310 | 339.1226 | 135.0451, 203.0711 | [4] |
| 14 | 9.37 | Kuwanon G | C_40_H_36_O_11_ | [M+H]+ | 692.2257 | 693.2316 | 137.0230, 203.0700, 365.1014, 299.0545, 421.1640 | [5] |
| 15 | 9.48 | Dihydrokuwanon S | C_25_H_28_O_5_ | [M-H]- | 409.1942 | 407.1859 | 137.0243 | [1] |
| 16 | 9.55 | Guangsangon C | C_35_H_30_O_10_ | [M-H]- | 610.1844 | 609.1762 | 499.1400, 337.1086, 227.0713 | [1] |
| 17 | 9.67 | Sanggenon N | C_25_H_26_O_7_ | [M-H]- | 438.1684 | 437.1603 | 368.0890, 151.0037, 125.0243 | [1] |
| 18 | 9.78 | Morusinol | C_25_H_26_O_7_ | [M-H]- | 438.1678 | 437.1603 | 125.0243, 283.1335 | [6] |
| 19 | 9.79 | Hydroxyisokuwanon G | C_40_H_38_O_12_ | [M-H]- | 710.2368 | 709.2250 | 473.1623, 125.0243, 109.0294 | [1] |
| 20 | 9.79 | Broussochalcone B | C_20_H_20_O_4_ | [M-H]- | 324.1361 | 323.1287 | 203.0712 | [4] |
| 21 | 9.83 | Kuwanon C | C_25_H_26_O_6_ | [M-H]- | 422.1735 | 421.1670 | 109.0296, 125.0243, 299.1286, 295.1333, 352.0945 | [1] |
| 22 | 9.93 | Sanggenon A | C_25_H_24_O_7_ | [M-H]- | 436.1522 | 435.1443 | 57.0346, 125.0243 | [6] |
| 23 | 10.06 | Sanggenol A | C_25_H_28_O_6_ | [M-H]- | 424.1885 | 423.1810 | 405.1692, 297.1494 | [2] |
| 24 | 10.09 | Dihydrorubraflavone A | C_25_H_28_O_5_ | [M-H]- | 408.1942 | 407.1869 | 389.1573, 297.1494, 109.0294 | [1] |
| 25 | 10.13 | Kazinol A | C_25_H_30_O_4_ | [M-H]- | 394.2144 | 393.2055 | 271.1702, 121.0295 | [4] |
| 26 | 10.15 | Kuwanon A | C_25_H_24_O_6_ | [M-H]- | 420.1578 | 419.1499 | 125.0243, 151.0035, 231.0660 | [1] |
| 27 | 10.16 | Sanggenol Q | C_25_H_28_O_6_ | [M-H]- | 424.1891 | 423.1812 | 395.1861, 379.1219, 125.0244 | [1] |
| 28 | 10.37 | Papyriflavonol A | C_26_H_28_O_7_ | [M-H]- | 452.1835 | 451.1766 | 436.1523 | [4] |
| 29 | 10.40 | Morusin | C_25_H_24_O_6_ | [M-H]- | 420.1572 | 419.1503 | 297.1133, 109.0291 | [6] |
| 30 | 10.44 | Isosanggenon J | C_25_H_26_O_6_ | [M-H]- | 422.1734 | 421.1647 | 269.1533, 151.0035, 125.0243 | [1] |
| 31 | 10.52 | Kuwanol D | C_25_H_28_O_5_ | [M-H]- | 408.1936 | 407.1834 | 255.1752 | [2] |
| 32 | 10.63 | Dihydrosanggenon V | C_25_H_26_O_6_ | [M-H]- | 422.1734 | 421.1648 | 295.1336, 151.0036, 125.0243 | [1] |
| 33 | 10.74 | Sanggenol D | C_30_H_36_O_7_ | [M-H]- | 508.2461 | 507.2354 | 437.1563 | [2] |
| 34 | 10.77 | Kuwanon S | C_25_H_26_O_5_ | [M-H]- | 406.1785 | 405.1701 | 321.0760, 282.0527 | [1] |
| 35 | 10.80 | Sanggenon J | C_25_H_26_O_6_ | [M-H]- | 422.1734 | 421.1650 | 151.0039, 125.0243 | [1] |
| 36 | 10.92 | Sanggenol H | C_30_H_36_O_6_ | [M-H]- | 492.2517 | 491.2430 | 365.2117, 125.0243 | [1] |
| 37 | 11.02 | Sanggenol P | C_30_H_36_O_6_ | [M-H]- | 492.2517 | 491.2420 | 463.2484, 193.0868, 177.0195 | [1] |
| 38 | 11.19 | Sanggenol B | C_30_H_34_O_6_ | [M-H]- | 490.2355 | 489.2278 | 261.1494, 471.2214 | [2] |
| 39 | 11.25 | 3'-Geranyl-3-prenyl-2';4';5;7-tetrahydro-xyflavone | C_30_H_34_O_6_ | [M-H]- | 490.236 | 489.2275 | 231.0655 | [1] |
| 40 | 11.26 | Cyclomorusin | C_25_H_22_O_6_ | [M-H]- | 418.1416 | 417.1337 | 373.1431 | [2] |
| 41 | 11.97 | Isosanggenol G | C_25_H_26_O_6_ | [M-H]- | 422.1734 | 421.1651 | 309.0417, 125.0241 | [1] |

**Reference**

[1] Q.Y. Zhou, X. Liao, H.M. Kuang, J.Y. Li, S.H. Zhang, LC-MS Metabolite Profiling and the Hypoglycemic Activity of Morus alba L. Extracts, Molecules, 27 (2022) 5360.

[2] W. Li, L. Chen, M. Li, K. Peng, X. Lin, Y. Feng, Y. Zou, X. Wu, Study on chemical composition, anti-inflammatory activity and quality control of the branch bark of Morus alba L. Fitoterapia. 181 (2025) 106383.

[3] Y. Zheng, E.H. Lee, S.Y. Lee, Y. Lee, K.O. Shin, K. Park, I.J. Kang, Morus alba L. root decreases melanin synthesis via sphingosine-1-phosphate signaling in B16F10 cells, J. Ethnopharmacol. 301 (2022) 115848.

[4] M.H. Park, S. Jung, H.J. Yuk, H.J. Jang, W.J. Kim, D.Y. Kim, G. Lim, J. Lee, S.R. Oh, S.U. Lee, H.W. Ryu, Rapid identification of isoprenylated flavonoids constituents with inhibitory activity on bacterial neuraminidase from root barks of paper mulberry (Broussonetia papyrifera), Int. J. Biol. Macromol. 174 (2021) 61-68.

[5] S. Han, H. Kim, M.Y. Lee, J. Lee, K.S. Ahn, I. J. Ha, S.G. Lee, Anti-Cancer Effects of a New Herbal Medicine PSY by Inhibiting the STAT3 Signaling Pathway in Colorectal Cancer Cells and Its Phytochemical Analysis, Int. J. Mol. Sci. 23 (2022) 14826.

[6] N. Li, X. Du, T. Qu, H. Ren, W. Lu, X. Cui, J. Hu, Z. Chen, H. Tao, Pharmacodynamic material basis and pharmacological mechanisms of Cortex Mori against diabetes mellitus, J. Ethnopharmacol. 324 (2024) 117781.
